# Pulmonary neuroendocrine carcinoma mimicking neurocysticercosis: a case report

**DOI:** 10.1186/s13256-016-0910-y

**Published:** 2016-06-02

**Authors:** John C. Lam, Stephen R. Robinson, Andrew Schell, Stephen Vaughan

**Affiliations:** 1 Department of Medicine, The University of Calgary, Calgary, AB T2N 2T9 Canada; 2 Department of Pathology and Laboratory Medicine, The University of Calgary, Calgary, AB T2N 2T9 Canada; 3 Department of Infectious Disease, The University of Calgary, Calgary, AB T2N 2T9 Canada; 4 Foothills Medical Center, The University of Calgary, 1403 29 Street NW, Calgary, AB T2N 2T9 Canada

**Keywords:** Neurocysticercosis, Brain, Cysts, Adenocarcinoma, Metastases

## Abstract

**Background:**

Neurocysticercosis occurs when the eggs of the pork tapeworm (*Taenia solium*) migrate and hatch into larvae within the central nervous system. Neurocysticercosis is the most common cause of seizures in the developing world and is characterized on brain imaging by cysts in different stages of evolution. In Canada, cases of neurocysticercosis are rare and most of these patients acquire the disease outside of Canada. We report the case of a patient with multiple intracranial lesions whose history and diagnostic imaging were consistent with neurocysticercosis. Pathological investigations ultimately demonstrated that her brain lesions were secondary to malignancy. Brain metastases are considered to be the most common cause of intracranial cystic lesions.

**Case presentation:**

We present the case of a 60-year-old Canadian-born Caucasian woman with a subacute history of ataxia, lower extremity hyper-reflexia, and otalgia who resided near a pig farm for most of her childhood. Computed tomography and magnetic resonance imaging showed that she had multiple heterogeneous intracranial cysts, suggestive of neurocysticercosis. Despite a heavy burden of disease, serological tests for cysticercosis were negative. This result and a lack of the central scolices on neuroimaging that are pathognomonic of neurocysticercosis prompted whole-body computed tomography imaging to identify another etiology. The whole-body computed tomography revealed right hilar lymphadenopathy associated with soft tissue nodules in her chest wall and abdomen. A biopsy of an anterior chest wall nodule demonstrated high-grade poorly differentiated carcinoma with necrosis, which stained strongly positive for thyroid transcription factor-1 and synaptophysin on immunohistochemistry. A diagnosis of stage 4 metastatic small cell neuroendocrine carcinoma was made and our patient was referred for oncological palliative treatment.

**Conclusions:**

This case illustrates the importance of the diagnostic approach to intracranial lesions. Our patient’s diagnosis of neuroendocrine carcinoma was delayed because of her nontraditional presentation. Despite extensive metastatic burden, the lack of perilesional edema and the identification of lesions appearing to be in various stages of development led to a pursuit of neurocysticercosis as the diagnosis. The absence of constitutional symptoms should not discount the possibility of malignancy from the differential diagnosis.

## Background

Cysticercosis occurs after the ingestion of eggs from *Taenia solium* (pork tapeworm). Passage through the gastrointestinal tract dissolves the coating around the eggs, thereby allowing the cystic larvae to disseminate to different sites in the body.

Neurocysticercosis involves the migration and development of such larvae within the brain or extraparenchymal tissue. Seizures, headache, and findings of increased intracranial pressure are common manifestations of neurocysticercosis. On radiography, neurocysticercosis is characterized by multiple intracranial cysts in different stages of development. While the incidence of the disease in Canada remains low, neurocysticercosis continues to be the most common cause of seizures in developing countries [1].

## Case presentation

A previously healthy 60-year-old Caucasian woman presented to our hospital with a year-long history of right-sided otalgia diagnosed as otitis media. Despite multiple courses of antibiotics, the symptoms persisted. Two months prior to admission, she complained of difficulty balancing and non-specific spontaneous involuntary movements in her lower extremities. She had no history of seizure or headache. She reported no constitutional symptoms or respiratory complaints. Her past medical history was significant for a 60-pack year smoking history.

Our patient was born in rural Western Canada and moved to Eastern Canada as a child. She resided with her family adjacent to a pig farm but did not work directly with livestock. She returned to rural Western Canada as a young adult and was living there at the time of admission. She has never travelled outside of North America. She had no family history of neurological conditions or malignancy.

A neurological examination (including a fundoscopy) demonstrated normal cranial nerves. Sensory and motor examinations were unremarkable. Examination of her reflexes revealed hyper-reflexia (without muscle spread) throughout her lower extremities. Bilateral ankle clonus was present (greater than six beats) along with fasciculation in her right hand. A positive Romberg’s sign and a wide-based ataxic gait were also noted. Cardiovascular and respiratory examinations were unremarkable and chest radiography performed prior to admission was normal.

**Fig. 1:**
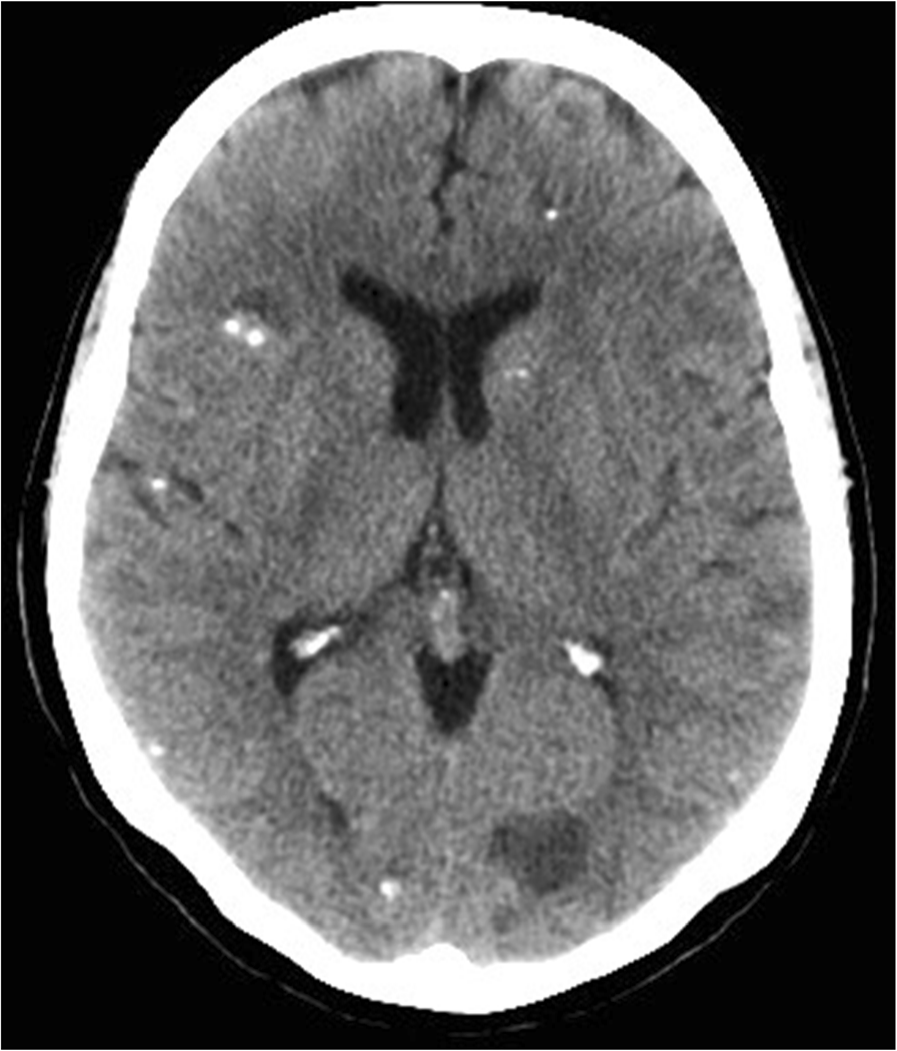
Computed tomography of the head demonstrated multiple bilateral well-defined cystic structures of different sizes associated with calcification without perilesional edema

Computed tomography (CT) of our patient’s head demonstrated multiple bilateral well-defined cystic lesions involving both her supratentorial and infratentorial regions. The cysts were mostly thin-walled of varying sizes, and some were calcified. No paralesional edema or mass effect was present (Fig. 1).

**Fig. 2:**
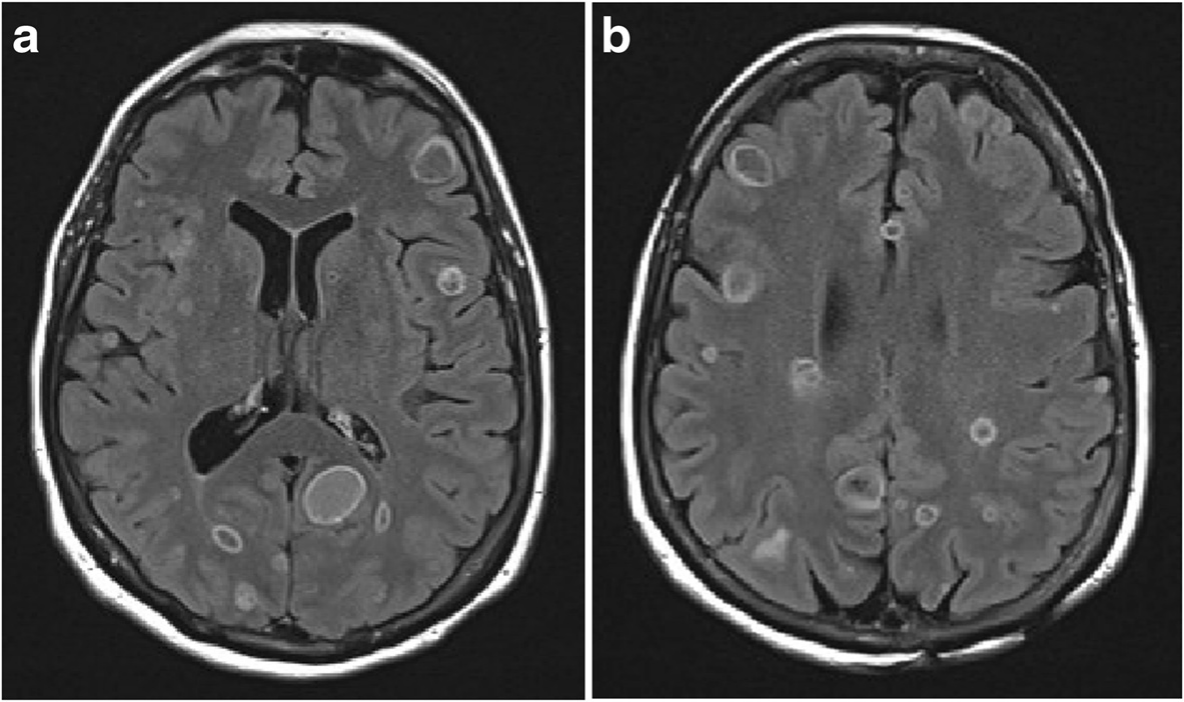
**a**, **b** Magnetic resonance imaging of the brain demonstrated multiple bilateral intracranial cystic lesions within the subarachnoid space and parenchyma. Differing signal intensities and fluids levels present on these cystic structures initially suggested neurocysticercosis in multiple stages of evolution

Magnetic resonance imaging of her brain with gadolinium contrast confirmed the presence of over 30 intracranial cystic lesions with differing fluid levels (Fig. 2a, b). Radiographic findings were consistent with multiple stages of neurocysticercosis evolution. However, no intra-lesional scolices or extraparenchymal involvement were seen. A skeletal survey was performed to assess for peripheral calcified lesions, with negative results. Our patient was diagnosed with presumptive neurocysticercosis and empirically started on albendazole, prednisone, and seizure prophylaxis while awaiting results from tests for *T. solium* serology.

Results of laboratory investigations, including renal function, hepatic enzymes, and cerebrospinal fluid analysis, were all within normal limits. When results of the cysticercosis serological tests came back negative despite an extensive burden of brain disease, whole-body CT was performed to check for other lesions that would be more amenable to biopsy.

Whole-body CT revealed right hilar enlargement (2 × 3 cm) associated with soft tissue nodules in her chest wall and abdomen. Equally concerning were infiltrates with irregular density along both adrenal glands and a low-density mass arising from the lateral capsule of her left kidney.

**Fig. 3:**
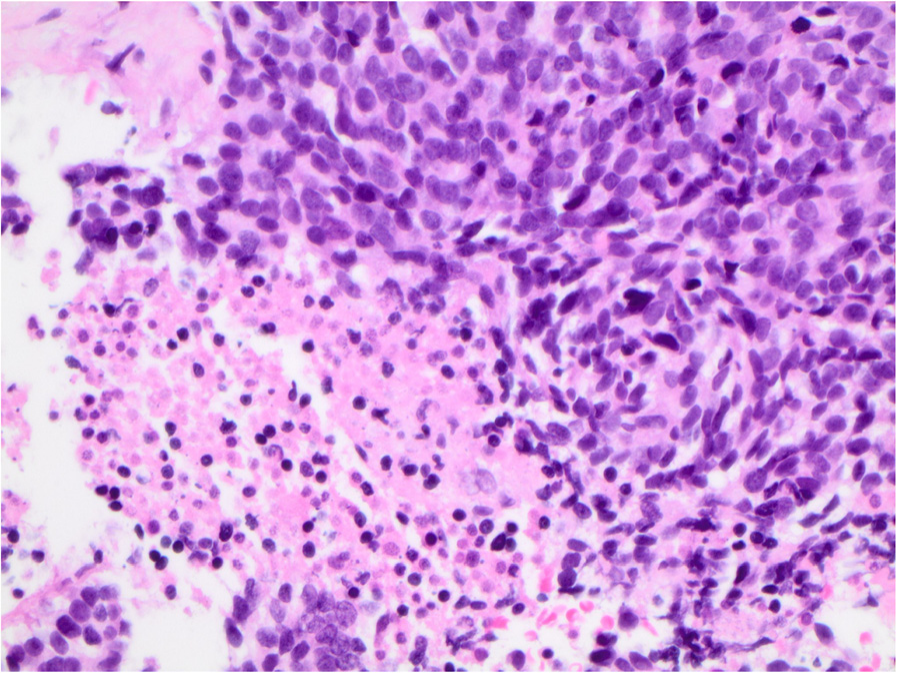
High power (400×) magnification of the chest wall biopsy confirmed dense crowding of neoplastic cells with small to medium sized overlapping and molded nuclei, and limited cytoplasm. Nuclear hyperchromasia with stippled chromatin lacking prominent nucleoli can be seen. An area of necrosis is well visualized in the lower left corner of the image

**Fig. 4:**
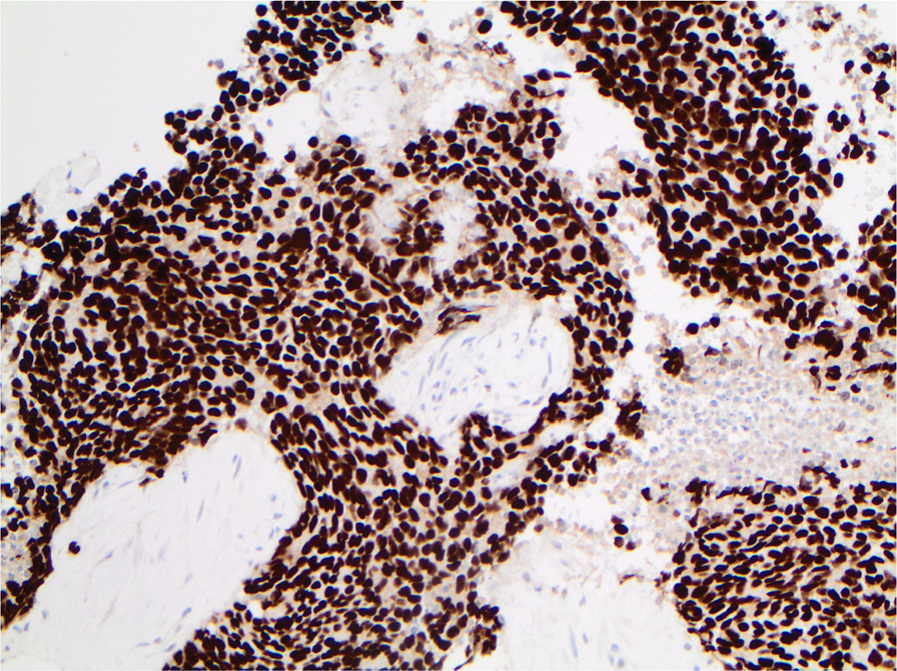
Neoplastic cells demonstrated strong diffuse nuclear positivity for thyroid transcription factor-1 (TTF-1) by immunohistochemistry (200× magnification). TTF-1 positivity is most frequently observed in neuroendocrine neoplasms of pulmonary origin

A biopsy of an anterior chest wall nodule demonstrated high-grade poorly differentiated carcinoma with necrosis (Fig. 3). The specimen stained strongly positive for thyroid transcription factor-1 and synaptophysin on immunohistochemistry (Fig. 4). A diagnosis of stage 4 metastatic small cell neuroendocrine carcinoma was made and our patient was referred for medical and oncological palliative radiation treatment. With palliative oncological therapies, her ataxia improved transiently but the spread of her malignancy continued, leading to her death 9 months after her original diagnosis.

## Discussion

Cysticercosis occurs after ingestion of eggs from *T. solium* (pork tapeworm). The coating around the eggs is dissolved by the gastrointestinal tract, allowing them to enter the bloodstream and be distributed to other tissues of the host. The cystic larvae (cysticerci) develop in multiple sites but are commonly found in the central nervous system (brain, spinal cord, ventricles) and skeletal muscle. Neurocysticercosis occurs when larvae migrate and develop in the brain or extraparenchymal tissue. Seizures and headache are usually associated with parenchymal cysts whereas extraparenchymal cysts are characterized by findings of increased intracranial pressure. Neurocysticercosis is the most common cause of seizures in developing countries [1].

Larvae remain viable for years, and multiple mechanisms of host immune tolerance have been postulated. While some larvae succumb to the host’s inflammatory reaction and form a granuloma in defense, other cysticerci do not. In the initial colloidal stage, the pathognomic finding of a cysticerci scolex within a thin-walled cystic lesion may be seen. In later stages, the walls thicken, calcify, and form a granuloma around the non-viable parasite. Imaging findings in neurocysticercosis differ depending on the stage of cyst development. Neuroimaging will commonly reveal multiple cysts in different developmental stages [2].

There is a broad range of etiologies for intracranial cystic lesions found on imaging (Table 1). Brain metastases are the most common cause of intracranial cystic lesions, with lung cancer being the most common primary tumor [3]. Clinically, only two-thirds of patients with brain metastases are symptomatic: headache and mental change are the commonest symptoms, and hemiparesis and altered mental status are the most frequent signs identified [4]. Classically, metastatic brain lesions appear as multiple solid enhancing masses that are well delineated with smooth borders and extensive edema. Mass effect and areas of central necrosis are common [5]. Smaller lesions may be associated with less perilesional edema.

**Table 1:** Differential diagnosis for intracranial cystic lesions [7, 8]

Malignant neoplasm: • Metastatic lesion • Primary brain neoplasm (central nervous system lymphoma, glioblastoma) Infection: • Bacterial (tuberculosis, pyogenic abscess, syphilis, nocardia, Whipple’s disease) • Endemic fungi (cryptococcus, coccidiomycosis, aspergillosis, histoplasmosis) • Parasitic (neurocysticercosis, cerebral sparganosis) • Viral (progressive multifocal leukoencephalopathy)	Inflammatory: • Multiple sclerosis • Acute disseminated encephalomyelitis • Transverse myelitis • Neuro-Behçet’s disease Other: • Neurosarcoidosis • Amyloidosis

It is important to consider metastatic disease when intracranial lesions are identified on imaging and to commence a workup for primary pathology. In situations of unknown but suspected primary malignant processes, a chest radiograph or CT of the chest is a reasonable starting investigation [6]. Our patient’s diagnosis of bronchogenic carcinoma was delayed because of her nontraditional presentation. Despite extensive metastatic burden, the lack of perilesional edema and the finding of lesions that appeared to be in various stages of development led to the pursuit of a neurocysticercosis diagnosis and empirical treatment. The absence of constitutional symptoms should not discount the possibility of malignancy from the differential diagnosis.

## Conclusions

Intracranial lesions have a broad differential diagnosis with brain metastases continuing to be the commonest cause in the developed world. Our case emphasizes the importance of a systematic approach when working up the etiology of intracranial lesions and of having a low threshold to investigate for malignancy.

## Abbreviation

CT: computed tomography

## References

[1] Garcia HH, Gonzalez AE, Evans CAW, Gilman RH, The Cysticercosis Working Group in Peru. *Taenia solium* cysticercosis. Lancet. 2003;362:547–56. 10.1016/S0140-6736(03)14117-7 PMC3103219 12932389

[2] Sarria Estrada S, Frascheri Verzelli L, Suirana Montilva S, Auger Acosta C, Rovira CA. Imaging findings in neurocysticercosis. Radiologia. 2013;55(2):130–41. 10.1016/j.rx.2011.11.009 22632836

[3] Norden AD, Wen PY, Kesari S. Brain metastases. Curr Opin Neurol. 2005;18:654–61. 10.1097/01.wco.0000191514.37498.2b 16280676

[4] Klos KJ, O’Neill BP. Brain metastases. Neurologist. 2004;1:31–46. 10.1097/01.nrl.0000106922.83090.71 14720313

[5] Loeffler JS, Patchell RA, Sawaya R. Metastatic brain cancer. In: DeVita VT, Hellman S, Rosenberg SA, editors. Cancer: Principles and Practice of Oncology. Philadelphia: Lippincott-Raven; 1997. p. 2523.

[6] Mavrakis AN, Halpern EF, Barker II FG, Gonzalez RG, Henson JW. Diagnostic evaluation of patients with a brain mass as the presenting manifestation of cancer. Neurology. 2005;65:908–11. 10.1212/01.wnl.0000176059.21455.76 16186533

[7] Garg RK, Desai P, Kar M, Kar AM. Multiple ring enhancing brain lesions on computed tomography: an Indian perspective. J Neurol Sci. 2008;266:92–6. 10.1016/j.jns.2007.09.012 17945258

[8] Omuro AM, Leite CC, Mokhtari K, Delattre JY. Pitfalls in the diagnosis of brain tumours. Lancet Neurol. 2006;5:937–48. 10.1016/S1474-4422(06)70597-X 17052661

